# A Regional Stable Carbon Isotope Dendro-Climatology from the South African Summer Rainfall Area

**DOI:** 10.1371/journal.pone.0159361

**Published:** 2016-07-18

**Authors:** Stephan Woodborne, Patience Gandiwa, Grant Hall, Adrian Patrut, Jemma Finch

**Affiliations:** 1 iThemba LABS, Private Bag 11, WITS, 2050, South Africa; 2 Discipline of Geography, School of Agricultural, Earth and Environmental Sciences, University of KwaZulu-Natal, Private Bag X01, Scottsville, 3209, Pietermaritzburg, South Africa; 3 Mammal Research Institute, University of Pretoria, Private Bag X20, Hatfield, 0028, South Africa; 4 Faculty of Chemistry, Babes-Bolyai University, Arany Janos 11, 400028, Cluj-Napoca, Romania; University of California Davis, UNITED STATES

## Abstract

Carbon isotope analysis of four baobab (*Adansonia digitata* L.) trees from the Pafuri region of South Africa yielded a 1000-year proxy rainfall record. The Pafuri record age model was based on 17 radiocarbon dates, cross correlation of the climate record, and ring structures that were presumed to be annual for two of the trees. Here we present the analysis of five additional baobabs from the Mapungubwe region, approximately 200km west of Pafuri. The Mapungubwe chronology demonstrates that ring structures are not necessarily annually formed, and accordingly the Pafuri chronology is revised. Changes in intrinsic water-use efficiency indicate an active response by the trees to elevated atmospheric CO_2_, but this has little effect on the environmental signal. The revised Pafuri record, and the new Mapungubwe record correlate significantly with local rainfall. Both records confirm that the Medieval Warm Period was substantially wetter than present, and the Little Ice Age was the driest period in the last 1000 years. Although Mapungubwe is generally drier than Pafuri, both regions experience elevated rainfall peaking between AD 1570 and AD 1620 after which dry conditions persist in the Mapungubwe area until about AD 1840. Differences between the two records correlate with Agulhas Current sea-surface temperature variations suggesting east/west displacement of the temperate tropical trough system as an underlying mechanism. The Pafuri and Mapungubwe records are combined to provide a regional climate proxy record for the northern summer rainfall area of southern Africa.

## Introduction

Radiocarbon dating of baobab (*Adansonia digitata* L.) trees in southern Africa has demonstrated the existence of a fused, multi-stem architecture that may be one of the reasons why some of these trees achieve ages in excess of 1000 years [1]. It has also been demonstrated that the carbon isotope (*δ*^13^C) values of the growth lamellae are a proxy for rainfall at the time of wood formation [2]. The development of a baobab-based rainfall proxy record for the last 1000 years for the Pafuri area of South Africa is important because of the scarcity of instrumental and proxy records for the region [3, 4]. The Pafuri record was used to contextualize the archaeological trajectory of the Iron Age in southern Africa [5] and also elucidate climate response to forcing over longer time scales than can be obtained from the instrumental record. It revealed that the Medieval Warm Period was relatively wet, the Little Ice Age was relatively dry, and that inter-annual variability in rainfall is linked to sea-surface temperature regimes in the Agulhas Current Core region and also the Indian Ocean Dipole Moment Index [2]. It suggested that the El Niño/Southern Oscillation Index (ENSO) was not a consistent driver of rainfall over the last millennium.

The use of stable light isotope analyses as an environmental proxy in baobabs is based on leaf-level fractionation of carbon isotopes (*δ*^13^C). In C_3_ plants the discrimination between the isotopic composition of ambient CO_2_ in the atmosphere and that assimilated by the tree is dependent on the internal leaf concentration of CO_2_ (*c*_*i*_) and the ambient atmospheric concentration of CO_2_ (*c*_*a*_) expressed as the ratio *c*_*i*_/*c*_*a*_ [6, 7]. The mechanisms that control the fractionation have been quantified [6], and in trees that are not water limited *c*_*i*_ is regulated by the photosynthetic rate, which is controlled by temperature and irradiance. In trees that are water limited it is regulated by stomatal diffusion, which is controlled by edaphic water availability, wind and relative humidity [7]. In southern Africa where rainfall is strongly seasonal, highly episodic, and where droughts are frequent [8] the dominant influence for non-riparian trees will be edaphic moisture availability driven by rainfall.

In order to produce an isotopic climate proxy record it is necessary to account for the isotopic fluctuations of atmospheric CO_2_, and in the last 1000 years there has been a substantial change in this, and *c*_*a*_ [9]. The influence of changing atmospheric carbon isotope values is a simple application of an offset determined from established records [9], but the changes in *c*_*a*_ yield a more complex physiological response from the trees. Increased CO_2_ in leaves with rising *c*_*a*_ facilitates faster carbon assimilation with reduced water transpiration during photosynthesis. This is an effective increase in intrinsic water use efficiency (iWUE) [10], and it has the potential to distort the environmental signal in the isotope record. Some tree iWUE responses are passive while others are active [11]. Passive trees regulate stomatal conductance so that the increase in *c*_*i*_ (*dc*_*i*_) matches the increase in *c*_*a*_ (*dc*_*a*_). Trees with an active iWUE response down-regulate the impact of elevated *c*_*a*_ by reducing stomatal conductance or by increasing photosynthetic activity. Changing iWUE in trees is the physiological mechanism underlying the use of stable carbon isotope ratios as a proxy for climate, but the distortion of the record by elevated atmospheric CO_2_ in recent centuries needs to be considered

Despite these methodological considerations, stable isotope climate reconstruction in trees has advantages over traditional ring widths in terms of the number of trees that need to be measured. It has been shown that reproducible isotope records from as few as 4 trees can yield a systematic high frequency (year-to-year) record [12], and up to 7 trees are required to reproduce low frequency (decadal to centennial) variability [13]. A more pragmatic approach is to emphasize the amplitude of changes in the tree isotope records. Large amplitude isotopic excursions require fewer replicates to be interpreted with confidence, while small excursions require more replicates [14]. Such analogous arguments inform the required sample population, but they cannot be extrapolated to southern Africa where environmental drivers may vary. Where a variety of forcing factors act simultaneously, for example temperature, irradiance, canopy cover, and moisture availability, the likelihood that tree isotopes respond to micro-environmental conditions increases and with it the intra-population variability. The summer rainfall region in southern Africa savanna experiences redundancy between forcing by rainfall, temperature and sunlight, and with no canopy cover affecting the microclimate, these factors seldom yield conflicting responses in different trees. The greatest theoretical variability in the environmental forcing of tree isotopes in southern Africa is the heterogeneity of rainfall.

Precipitation in the summer rainfall region of Southern Africa derives mostly from convective storms [15], and the distribution of rainfall is extremely patchy. Isolated or scattered storms form over areas of approximately 200 km × 50 km and one third of the cells may coalesce into multicellular storms while <10% form squall lines [16]. Less than 25% of storm tracks cover >100 km^2^ and only 8% endure for more than an hour [17]. In addition, there is a strong E/W rainfall gradient. Although temperate tropical troughs (TTT) that are responsible for the convective cloud belt that brings the majority of the summer rainfall to the region [15] are typically orientated in a NW/SE orientation across the subcontinent [18], the system displaces along an E/W axis and there is a strong reduction in rainfall from the Mozambique coast in the east moving into the Limpopo River Valley [8], culminating in the arid Kalahari Desert in the west. Woodborne et al. [2] proposed that the westward (eastward) displacement of the TTT system was associated with cooler (warmer) sea-surface temperatures (SST) in the Agulhas Current region. Rainfall heterogeneity expresses at both local and regional scale, and multiple trees must be analyzed to account for this.

The reproducibility of the baobab isotope record is further contingent on an accurate age model for the proxy, and the Pafuri chronology used 17 radiocarbon dates, cross correlation of the climate record, and on two of the trees, ring structures that were presumed to be annual. The ability to stop growing for up to 500 years [1, 2] meant that the sampling depth in the Pafuri record varied between 1 and 4 trees.

The paucity of climate records that are sufficiently resolved in time and space has undermined the ability to discern synoptic shifts in response to long term forcing in southern Africa, as well as the cultural adaptation to these changes. Climate reconstructions from as few as 2 trees [19] have been widely referenced. Although the Pafuri record derives from 4 trees it may be questioned if it is representative of the broader climate variability in the region because of the heterogeneity in the climate system as well as the assumptions underlying the age model. In order to test this we analyzed additional baobab trees from the Mapungubwe region about 200 km to the west of Pafuri. The specific objectives were to establish an independent record of climate change at Mapungubwe because of the important archaeological heritage in the area; to verify the assumption that rings are annually formed in baobabs; and to account for the effect of changing iWUE that was not done for the Pafuri record. By comparing a climate record from Mapungubwe with that from Pafuri it is also feasible to test if the E/W displacement of the TTT rainfall system is driven by changes in SST in the Agulhas Current.

## Materials and Methods

### Baobab sampling

**Fig 1:**
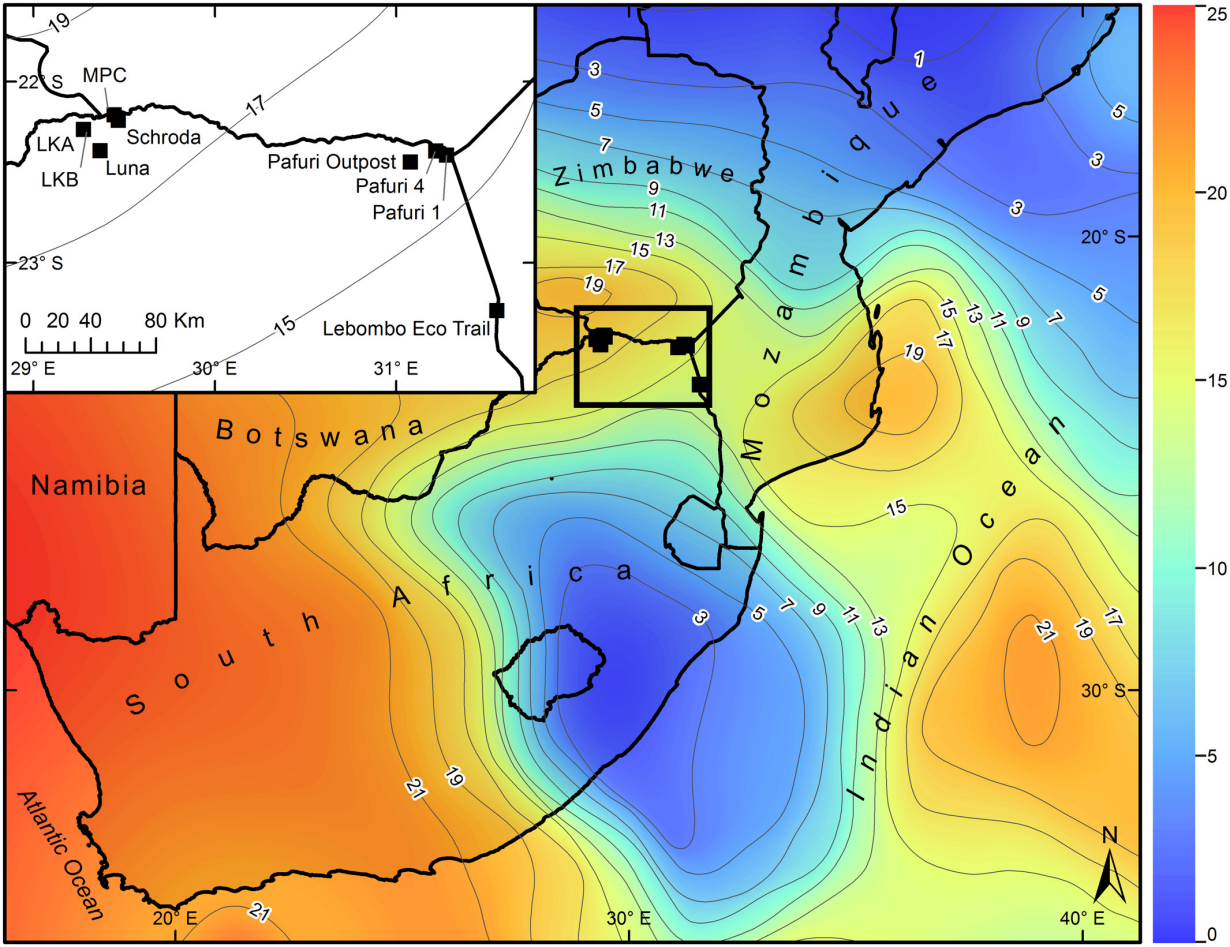
Map of the Mapungubwe baobab sample sites. The 5 baobab trees comprising the Mapungubwe sample came from approximately 200km west of the Pafuri baobab samples. The contour and color scale in the main map is an indication of the frequency of austral summer dry spells after. The color and contours are modified from [7] under a CC BY license, with permission from Inter Research Climate Research, original copyright 2004. The inset map indicates the relative proximity of individual trees to one another in the Mapungubwe and Pafuri records.

Five baobab trees were sampled in the vicinity of Mapungubwe (Fig 1), four of which were located within the Mapungubwe National Park, with the final tree located on De Beers private property. The South African National Parks is the designated authority approving research on baobabs within National Parks, and this work was done with their approval. The sampling of the tree on De Beers private property was done with their explicit permission. The first tree was designated the Leokwe A baobab (LKA) (22° 15.834'S, 29° 16.614'E, 579 m a.s.l.) which fell some time between 2003 and 2008 when it was found toppled but still intact. Permitting issues meant that the sampling could only take place in 2013 when decomposition was well advanced. The second tree was designated the Leokwe B baobab (LKB) (22° 15.803'S, 29° 16.647'E, 581 m a.s.l.) from a little more than 100m to the east of Leokwe A on the same rocky outcrop. The reason for the death of this tree is unknown but it was discovered with the base of the stem still in an upright position, with the upper structures missing. It is likely that the tree was killed by elephant damage but the section that was sampled retained the full record of growth rings from the bark to the center. The third tree was designated the Mapungubwe C baobab (MPC) (22° 12.847'S, 29° 28.084'E, 581 m a.s.l.). This was a small tree that fell over in 2014. Individual growth rings were the unit of analysis in these three dead trees, with the assumption that rings would be annual [2]. In addition, two trees were sampled during the field campaign of Patrut et al. [1] and Woodborne et al. [2] in 2010. These were the Schroda baobab (22° 10.996'S, 31° 26.716'E, 531 m a.s.l.) and the Luna baobab (22° 22.830'S, 29° 22.065'E, 664 m a.s.l.). The two standing baobabs were sampled with a Haglöf CO600 increment borer (60 cm long, 0.43 cm inner diameter) using the methodology described by Patrut et al. [20, 21].

### Baobab chronology

The chronology for the isotopic analysis of the baobabs followed the protocol of Woodborne et al. [2]. The cores from the Luna baobab and the Schroda baobab were subdivided into equal sized aliquots for the isotopic analysis. An age was attributed to each aliquot on the basis of a linear correlation between calibrated AMS radiocarbon dates and the aliquot number. The 1cm sections of the cores used in the AMS analyses were forfeited from the isotopic analysis. For the LKA, LKB the MPC baobabs the AMS radiocarbon dates were performed on individual rings, and there was sufficient material available that the isotopic analysis of these trees is complete. All of the AMS radiocarbon dates were calibrated using the 2013 Southern Hemisphere calibration dataset [22] and the Queens University, Belfast, online calibration programs version 7.0 (http://calib.qub.ac.uk/calib/calib.html) and the Calibomb program (http://calib.qub.ac.uk/CALIBomb/) which uses the bomb carbon dataset of Hua et al. [23] for the results with >100 percent modern carbon.

Robertson et al. [24] and Woodborne et al. [2] found the ring structures in baobabs from the eastern part of the subcontinent to be annually formed, but this was not the case for the Mapungubwe baobabs. The MPC baobab yielded 446 rings, while two AMS radiocarbon dates demonstrate that the tree was unlikely to have been older than 110 years, yielding an average of approximately 4.1 rings/year. The AMS radiocarbon dates for the LKA and LKB baobabs also indicated that these trees yielded an average of 1.3 and 0.6 rings/year, respectively. The emphatic demonstration that the baobab trees in the Mapungubwe area do not grow annual rings meant that the age model for the isotopic analysis could not rest on this assumption. Instead, the age model was constructed in the same way as it was for the cores, treating each successive ring as an aliquot and applying a linear regression to the ring count versus age plot.

**Fig 2:**
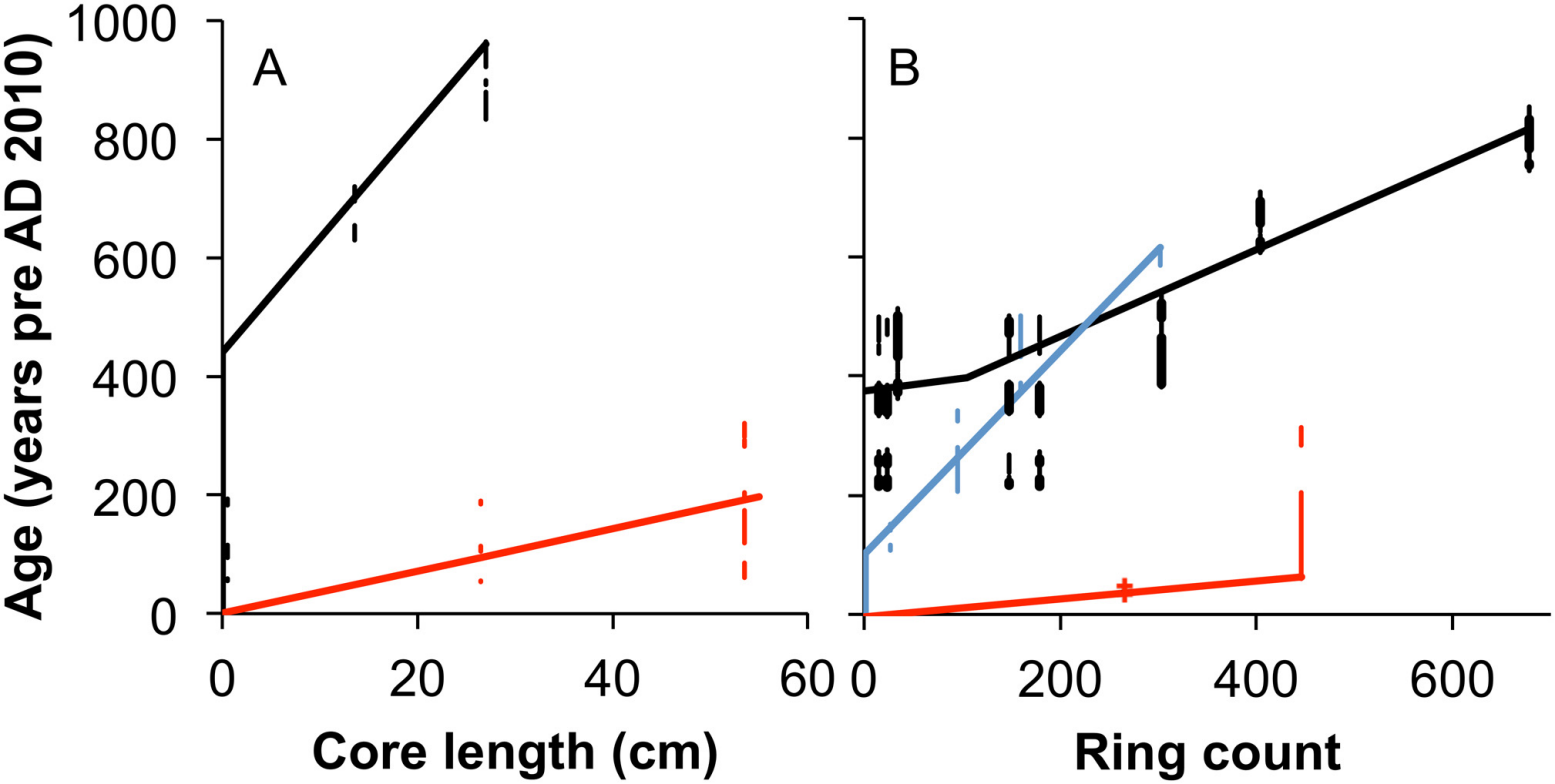
Age models for the baobabs. (A) Age models for the Schroda (red) and Luna (black) baobabs are based on incremental subsamples along the core. (B) The unit of analysis for the LKA (black), LKB (blue) and MPC (red) baobabs was rings. Vertical lines represent the one sigma AMS radiocarbon calibration ranges except for LKA where the two sigma range is represented, with the one sigma range represented as bold vertical lines. Crosses represent bomb-carbon AMS dates.

The ages used in the regressions were initially set as the most probable calibrated date for each AMS analysis, but subsequently adjusted within the one-sigma error range in order to modulate the resulting isotope time series between trees [2]. This approach worked for the age models from the LKB, MPC, Luna, and Schroda baobabs and all of the assigned ages fall within the one-sigma calibration range of the AMS radiocarbon dates. The approach did not work on the LKA baobab where a linear age model cannot pass through the one-sigma calibration ranges of all of the AMS dates. Producing an age model for this baobab was challenging and an exhaustive set of permutations were considered. By relaxing the requirement that assigned ages are within the 1-sigma range, a linear age model for rings 106 through 678 yielded a good isotopic correspondence with the Luna baobab and the older portion of the LKB baobab. However, this required that 3 of the 4 AMS radiocarbon dates from this section of the tree be assigned ages at the 2-sigma confidence level. For the most recent 105 rings the calibrated AMS dates produced an age inversion. The dates for ring 34 (340±30) and ring 145 (280±30) only overlap at the 2-sigma confidence range. Even if the 2-sigma calibration ranges are used this implies extremely rapid formation of the most recent 105 rings. Two potential scenarios may explain this: first, the rapid growth may be the result of buttress formation in which 105 rings were formed in approximately 20 years (c.5 rings/year compared with c.1.3 rings per year in the earlier growth of the tree), or second, this pattern is consistent with the fused multi-stem structure of baobabs [1], and the presence of a second stem may be indicated. Either scenario can be supported by the available AMS radiocarbon dates, but the buttress model was selected on the basis of the isotopic pattern-match with the LKB baobab. Whether this is correct is of little consequence as the different scenarios yield an almost identical isotope time series. The AMS radiocarbon dates are presented in Table 1, while the age models that were used are presented in Fig 2.

**Table 1:** Radiocarbon dates.

Tree	Core/Ring No.	Length (cm)*	Sample duration (years)	Date (Uncalibrated years BP)	Laboratory No.	Assigned year (AD)
LKA	15	Not noted	<1	280±30	Beta-378949	1632
	24	Not noted	<1	270±30	Beta-378950	1630^¶^
	34	Not noted	<1	340±30	Beta-378951	1628
	148	Not noted	<1	290±30	Beta-409150	1581^¶^
	179	Not noted	<1	280±30	Beta-384994	1558^¶^
	303	Not noted	<1	380±30	Beta-409151	1468^¶^
	404	Not noted	<1	650±30	Beta-380707	1394
	678	Not noted	<1	880±30	Beta-378952	1195
LKB	27	Not noted	<2	110±30	Beta-380705	1864
	95	Not noted	<2	210±30	Beta-409149	1747
	137	Not noted	<2	340±30	Beta384995	1636
	279	Not noted	<2	590±30	Beta-380706	1394
MPC	266	Not noted	<1	133.2±0.3pmc	Beta-409148	1974
	446	Not noted	<1	110±30	Beta-409147	1947
Luna	4	7.5	2.4	64±29	OS-86100	1922
	4	18.5	2.4	709±29	OS-86099	1310
	4	25.5	2.4	975±30	OS-78685	1050
Schroda	1	26.5	<1	26±25	OS-84392	1938
	1	53.5	<1	158±24	OS-90686	1866

^¶^Intercept at two-sigma confidence level

*Length measurements along cores originate at the surface of the trees

### Isotope analysis

Aliquots were pre-treated to α-cellulose [25] and the carbon isotopes measured at the Stable Light Isotope Laboratory, University of Pretoria, South Africa, on a DeltaV isotope mass spectrometer coupled with a Flash EA 1112 series elemental analyser by a Conflo IV. In-house wood standards (*Shorea superba*) [2, 26, 27] and blanks were run at the start and end of each analysis, and after every 12 unknown samples, and the precision of standards was <0.2‰. The isotopic time series from each baobab was adjusted for temporal changes of *δ*^13^C in the atmosphere [20], with updates available online (http://cdiac.ornl.gov/ftp/db1014/isotope.cgo).

The minimum length of aliquots was determined by the state of preservation of the cores, and the anticipated sample-loss during the pretreatment process. The average aliquot was 1.13mm in length for the Luna baobab core, and 1.46mm for the Schroda core. For the LKA, LKB and MPC baobabs the ring widths were not measured. The corresponding age stride of aliquots (years per aliquot) was 2.2 years, 0.3 years, 0.8 years, 1.6 years, and 0.2 years for the Luna, Schroda, LKA, LKB and MPC baobabs respectively.

A normalization step was undertaken to compensate for the physiological responses of individual trees to their edaphic conditions. On average the isotopic values from the Mapungubwe baobabs are -0.76‰ relative to the Pafuri record indicative of generally drier conditions. The Mapungubwe baobab with the closest offset is the LKB baobab, with a normalization factor of -0.71‰ relative to the Pafuri baobabs. This tree also has the broadest temporal coverage and for these reasons it was used as the reference against which the other Mapungubwe trees were normalized. The differences in isotopic values for overlapping sections of the different trees and LKB were applied as a constant correction for individual trees. The normalization resulted in correction factors of 1.42‰, 0.66‰, -0.58‰, 0‰, and -0.28‰ for the Luna, Schroda, LKA, LKB and MPC baobabs respectively.

Although the age stride for aliquots was variable and in some trees represents more than a single year, each aliquot was assigned a mean age from the model. Thereafter, the isotopic time series for each of the 5 baobabs were averaged annually to yield a single time series.

### Water use efficiency

The relationship between the measured carbon isotope ratio (*δ*^13^C_*plant*_) and the ambient atmospheric carbon isotope ratio (*δ*^13^C_*air*_) is give by the Farquar et al. [6] equation:

$$\delta^{13}C_{plant}=\delta^{13}C_{air}-\left(a+\left(b-a\right)\frac{c_{i}}{c_{a}}\right)$$ (1)

where *c*_*a*_ and *c*_*i*_ represent the atmospheric and plant intercellular CO_2_ concentrations respectively, *a* is the fractionation associated with stomatal diffusion (4.4‰) and *b* is the fractionation associated with carboxylation during photosynthesis (27‰). The value of *δ*^*13*^*C*_*air*_ and *c*_*a*_ are known for the last 1000 years [9] and accordingly it is possible to calculate *c*_*i*_ for any known-age wood for which *δ*^13^C has been measured. Wang & Feng [11] define the term *Φ* as:

Φ=dcidca (2)

and demonstrated that *Φ* is approximately constant for any tree. The term *Φ* reflects the physiological response of the tree to elevated concentrations of atmospheric CO_2_ and it ranges from *Φ* = 1 (passive response) to *Φ* = 0 (constant response). The value of *dc*_*i*_ and *dc*_*a*_ were calculated by subtracting the average value of ci (*c*_*a*_) for the period pre AD 1795 before atmospheric CO_2_ concentrations increased, from the period post AD 1900. By averaging these values over periods of centuries the high frequency variability is suppressed and the low frequency variation that represents the plant’s response to elevated CO_2_ [11] is captured. Three of the baobab samples from Pafuri and Mapungubwe span the pre AD 1795 and the post AD 1900 period, and the average *Φ* value was calculated for these trees.

The term *Φ* can be substituted into Eq (1) so that:

$$\Delta^{13}C_{iWUE}=\delta^{13}C_{air\;pre\;1795}-\left[\delta^{13}C_{air}-\left(a+\left(b-a\right)\frac{c_{i\;pre\;1795}-\Phi dc_{a}}{c_{a}}\right)\right]$$ (3)

where *Δ*^13^C_*iWUE*_ is the change in *δ*^13^C_*plant*_ that can be attributed to changes in water use efficiency. The approach has an inherent assumption that *Φ* reflects a response to changes in iWUE, and not to mesophyll conductance or photorespiration, and it also presumes that there is no significant change in the environmental forcing between the pre AD 1795 period and the post AD 1900 period. The Pafuri record suggests drier conditions in the pre AD1795 period, but the overall effect of changing iWUE in response to elevated CO_2_ will be shown to have a relatively small effect on the isotope proxy record, and the change in rainfall can also be shown to be irrelevant in the calculation.

### Statistics

Woodborne et al. [2] discuss the errors in this approach to age modeling baobab growth. Primary errors lie in the latitude in the AMS radiocarbon dating calibration, and secondary errors lie in the fact that each aliquot represents a different time interval. In addition, the assumption that radial growth is linear will introduce age underestimations during periods of accelerated growth, and age overestimations during periods of slow growth. The age models used to produce the Pafuri record were based on the combined errors of multiple AMS radiocarbon dates and it was estimated that the error in the age of any individual aliquot was in the order of ±5 years. The age models for the Mapungubwe trees required 5 of the 18 AMS radiocarbon dates to be assigned at the 2-sigma range. In terms of a probability distribution of age estimates this is not extraordinary, as the accuracy of AMS radiocarbon dates should follow a normal distribution, with 67% of age estimates being accurate within 1-sigma error estimates. However, all 5 of the ages assigned at the 2-sigma level were from the LKA baobab. The growth of this tree appears idiosyncratic, but the isotopic record matches the record from the other Mapungubwe trees which are temporally well constrained. The estimated error is similar to that achieved in the Pafuri study.

The high frequency *δ*^13^C variability within each tree profile is driven by high rainfall heterogeneity in the summer rainfall region of southern Africa. In order to suppress the local rainfall variability and extract temporal changes that represent climate variability (rather than weather variability), Woodborne et al. [2] calculated a 21-year biweight mean statistic [28] for the Pafuri record. The 21-year biweight mean calculates a scaled weighted mean *δ*^13^C value incorporating the 10 years before and the 10 years after the year for which the calculation is being made. The statistic suppresses the high frequency component of the record, and emphasizes the non-stationary component [2]. A 21-year biweight mean statistic using a control factor of 9 was calculated for the Mapungubwe *δ*^13^C record.

Time series comparisons were calculated between the biweight mean *δ*^13^C values from the Pafuri and Mapungubwe baobabs. The combined record from the two areas was then compared with the climate forcing variables explored by Woodborne et al. [2]. The Climate Explorer website (http://climexp.knmi.nl) that uses a Monte Carlo method to calculate the confidence ranges, and takes autocorrelations into account in calculating p-values, was used for the calculation of confidence statistics.

## Results and Discussion

### Ring structure

Previous age modeling of baobab rings [2, 24] showed that radiocarbon dates and ring counts for baobabs in the Kruger National Park were consistent with annual ring formation. A surprising outcome of the age modeling for the Mapungubwe baobabs is the unambiguous demonstration that these trees do not form annual rings. For three of the trees in the current study the isotopic analysis were on rings (not equally spaced aliquots), and the AMS radiocarbon dating shows that the averaged ring frequency varies between 0.6 rings/year (LKB baobab) to 4.1 rings/year (MPC baobab). The ring structures are very distinct and there is little chance that mistaken identifications could be the cause of such a substantial mismatch between rings and years. The Mapungubwe region (mean rainfall = 337mm between 1955 and 2005) is drier than the Pafuri region (mean rainfall = 433mm between 1955 and 2005) and is subject to more frequent dry spells (defined as less than 5mm of rainfall in a period of 5 consecutive days during the rainy season) [8]. It is possible that edaphic moisture frequently drops below a critical threshold for baobab photosynthesis. It has been shown that baobabs retain high stem water content, but this is not used to buffer leaf-level water deficits [29, 30]. In addition they are physiologically drought adapted and they enter a state of dormancy in which water transpiration at leaf level, and water uptake at root level is negligible [31]. The dormancy of baobabs appears to be extreme, extending to several decades or even centuries [1, 2]. The combination of frequent dry periods in the Mapungubwe region, and the dormancy capability of baobabs, may suggest that growth in this region is strongly event driven, and rings do not represent annual cycles in edaphic soil moisture, but rather sub-seasonal rainfall events.

That baobab growth rings are not necessarily annually formed implies that the age model should be constructed on the basis of the radiocarbon dates and climate record coherence alone. In the Pafuri record two trees were assumed to have annual rings, and these were reassessing using the revised criteria. Two of the dates on the Pafuri Gila Pan baobab are re-interpreted with amendments of 54 years and 18 years. Both of these adjustments are within the 1-sigma error ranges of the radiocarbon dates and the revised age model removes a hiatus from the previous age model. There was no evidence to suggest that the age model for the Pafuri Outpost Baobab, the second tree in the Pafuri record for which rings were presumed to be annual, be amended.

### Water use efficiency

The value obtained for *Φ* was 0.43 ppmv*c*_*i*_/ppmv*c*_*a*_ (range: 0.37–0.52), which is very similar to the value of 0.46±0.02 ppmv*c*_*i*_/ppmv*c*_*a*_ obtained across a range of species by Wang & Feng [11]. This is indicative of an active down-regulation of *c*_*i*_ in response to increasing *c*_*a*_ by baobabs. The effect applies after AD 1795 when *c*_*a*_ began to increase, but it becomes more pronounced in the last 50 years. Using Eq (3) the records for Mapungubwe and Pafuri were corrected. The scale of the correction is dependent on *c*_*i pre 1795*_, and amounts to approximately +0.63‰ in AD 2000.

### Mapungubwe rainfall proxy record

**Fig 3:**
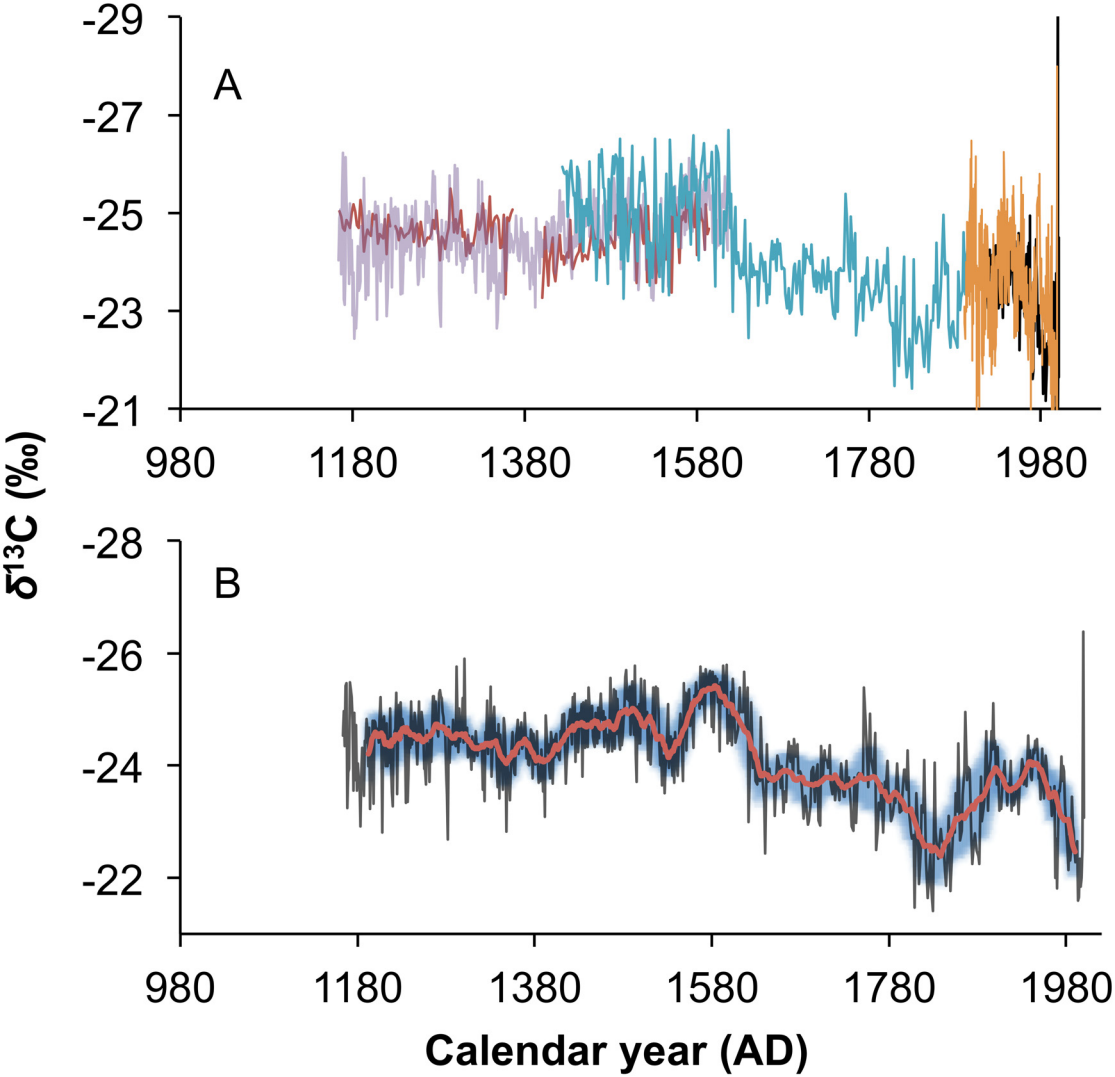
The baobab *δ*^13^C record from Mapungubwe. (A) The isotopic record from individual trees from Mapungubwe: LKA (purple), LKB (blue), Luna (red), MPC (orange) and Schroda (black). (B) The record from the 5 baobabs in the in the Mapungubwe sample are averaged (black) and the 21-year biweight mean (red) is calculated. The blue shading portrays the 21-year biweight mean variance.

The isotopic time series for the Mapungubwe baobabs is presented in Fig 3. The averaged *δ*^13^C record, and the 21-year biweight mean record from the Mapungubwe baobabs are also presented. The historic portion of these records can be compared with the instrumental rainfall record to verify the use of *δ*^13^C as a rainfall proxy.

**Fig 4:**
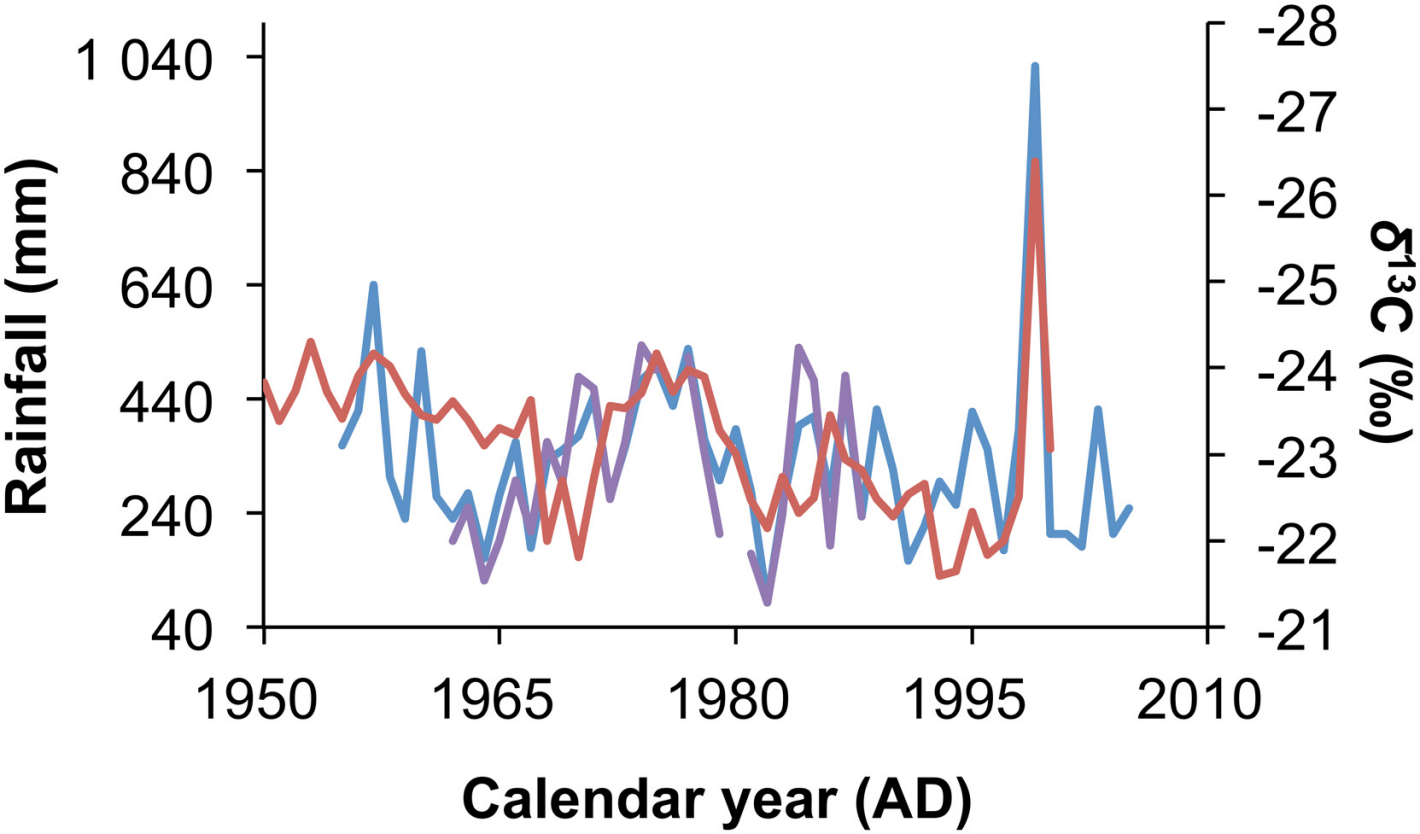
Rainfall calibration of the Mapungubwe baobab *δ*^13^C record. Instrumental rainfall records from Beitbridge provided by the Zimbabwe Weather Service (blue, left axis) and the South African Weather Service (purple, left axis) are significantly correlated with the *δ*^13^C record from the Mapungubwe baobabs (red, right axis).

Two instrumental rainfall records are available for the Mapungubwe area: a continuous monthly rainfall record from the Zimbabwe Weather Service at Beitbridge border post (approximately 55km east of Mapungubwe) from 1955 to 2005. This is confirmed with a partial record from the South African Weather Service from the same location between 1962 and 1968. The correlation between these datasets is highly significant (r = 0.767, P<0.001, n = 50). The Zimbabwe Beitbridge rainfall record is used for the baobab comparison because it is more complete. This yields a significant correlation (r = -0.576, p<0.001, n = 46) confirming the previous evidence that the baobab *δ*^13^C record is a proxy for rainfall (Fig 4). The revised chronology for the Pafuri baobabs also yields a significant correlation between *δ*^13^C and the instrumental record (r = -0.323, p<0,005, n = 82), which is stronger than that reported using the previous chronology (r = -0.246, p = 0.024, n = 84) [2].

**Fig 5:**
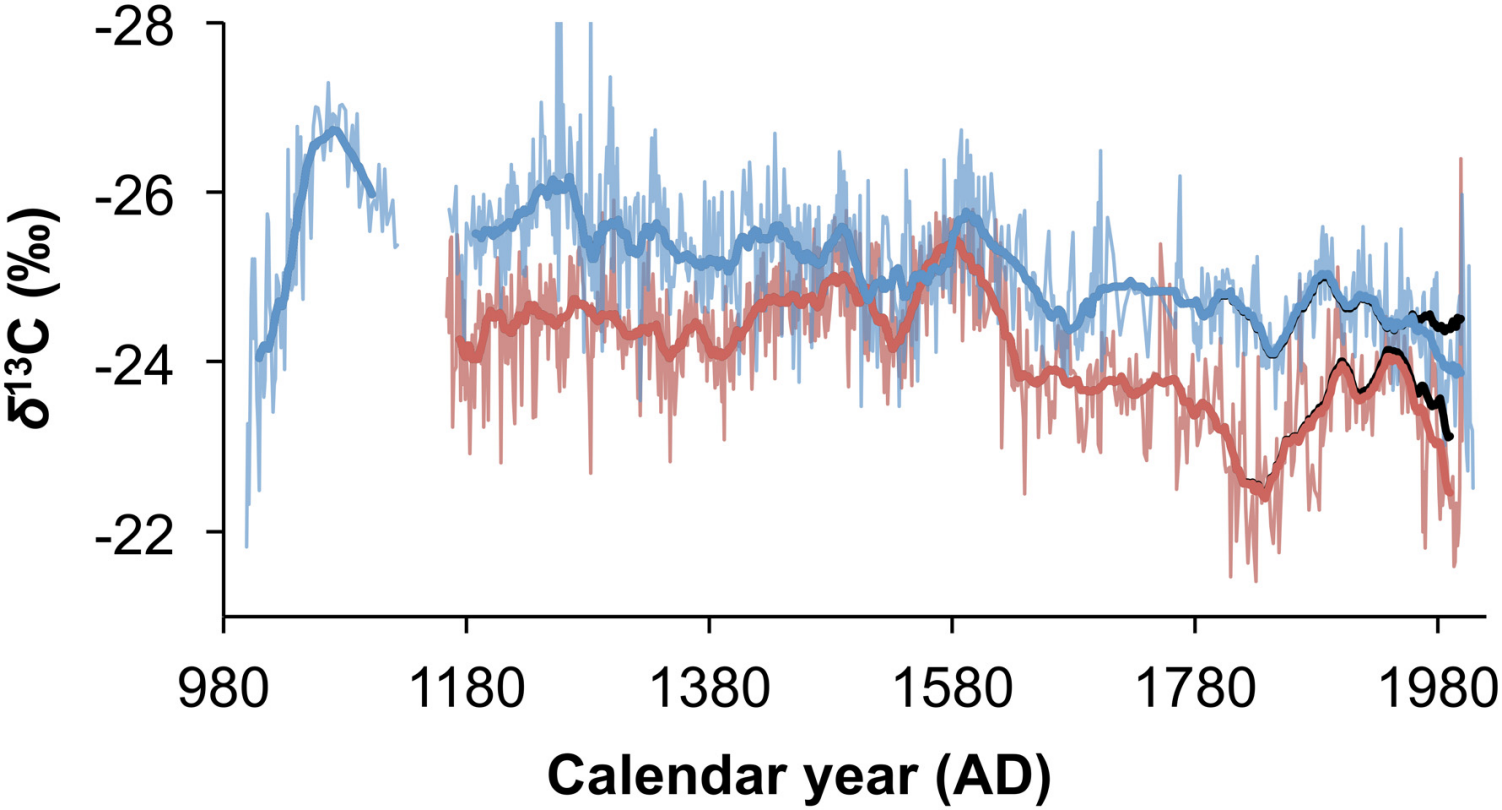
Comparing the Mapungubwe and Pafuri *δ*^13^C records. The average Mapungubwe *δ*^13^C record (red) and biwieght mean (bold red) has similarities and differences when compared with the Pafuri *δ*^13^C record (blue) and biwieght mean (bold blue). The black lines represent the biweight mean *δ*^13^C records from Mapungubwe and Pafuri without correcting for the intrinsic water use efficiency effect caused by elevated atmospheric CO_2_ concentrations since AD 1795.

The rainfall proxy record for Mapungubwe is presented in Fig 5 with the revised record from Pafuri. The Mapungubwe and Pafuri records are similar to one another (r = 0.705, p<0.001, n = 635) as may be expected because of their close proximity, but there are also differences that reflect localized effects.

### Synoptic shifts

The Pafuri rainfall proxy record was used to infer climate forcing in the region, and high frequency (decadal to seasonal) variability was linked to sea-surface temperatures (SST). When the Mapungubwe record is compared with sea-surface temperatures in the Agulhas Current core region [32, 33] the relationship is significant prior to AD 1940 (r = 0.477, p<0.001, n = 189) but is weaker during the latter part of the 20^th^ Century. The relationship with the Indian Ocean Dipole Moment Index [34] is weak (r = -0.062, p = 0.513, n = 114). The correlation with the El Niño Southern Oscillation (ENSO) proxy [35] is weak prior to AD 1650 (r = 0.214, p0.262, n = 322), but stronger after the Little Ice Age (post-AD 1750: r = 0.532, p<0.05, n = 188).

**Fig 6:**
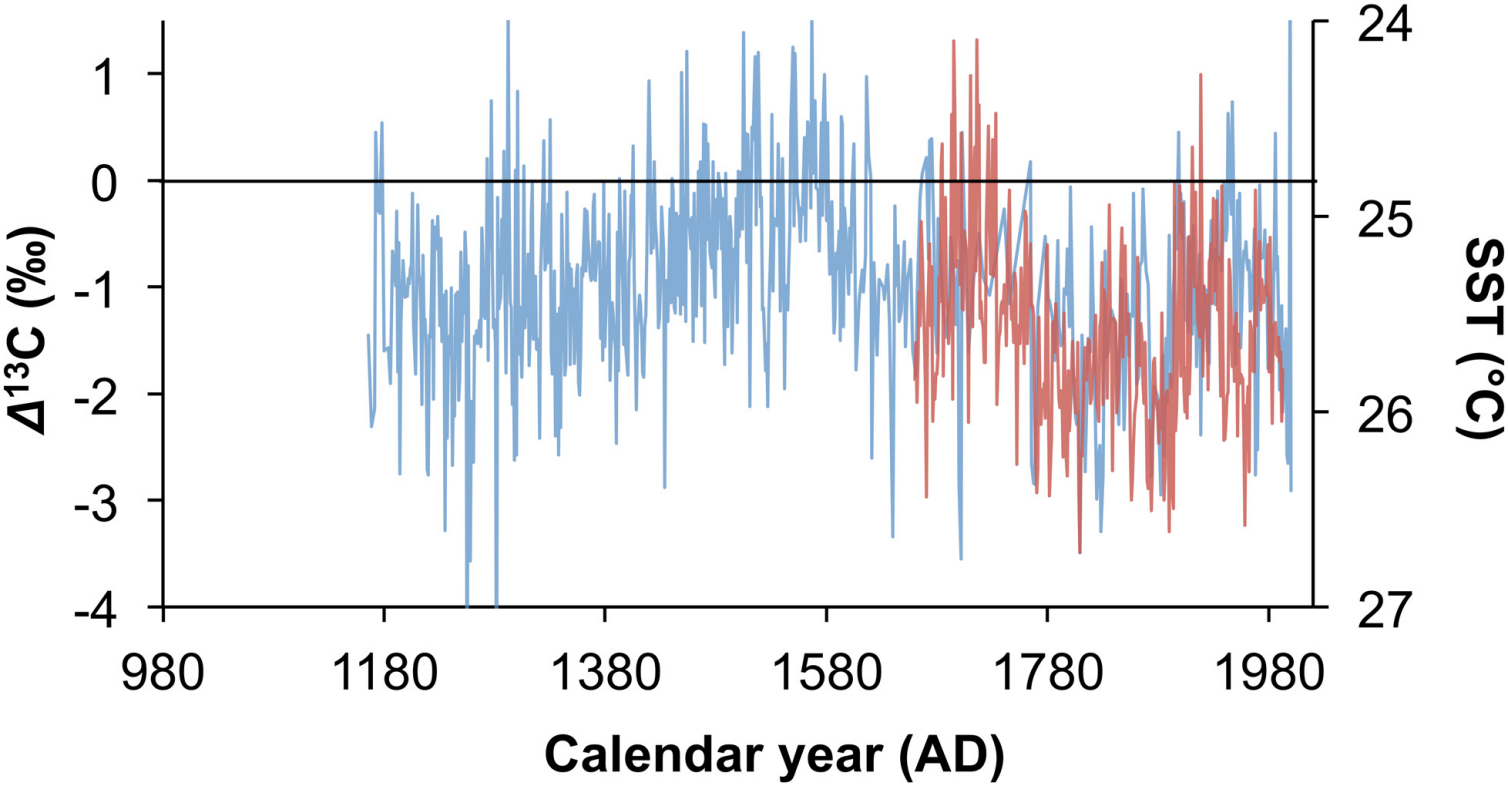
Residuals between the Mapungubwe and Pafuri *δ*^13^C records. The generally negative residual between the Mapungubwe record and the Pafuri record is caused by a lower rainfall in the Mapungubwe region (blue, left axis). This correlates significantly with the sea surface temperatures in the Agulhas Current Core region [32, 33] (red, right axis), suggesting that the residual is forced by the east/west location of the temperate tropical trough rainfall system.

It was suggested that the differences between the Mapungubwe and Pafuri records reflect localized effects, and this aligns with the suggestion that the Agulhas Current SST had a strong influence on the E/W position of the temperate tropical trough (TTT) cloud band that is associated with the dominant rainfall in the region [2]. The residuals between the Pafuri and Mapungubwe records (Fig 6) show that the rainfall differential between the two areas is patterned. At about AD 1250 Mapungubwe experienced conditions that were relatively drier than Pafuri, but there is a gradual wetting until approximately AD 1500 to AD 1600 when the two regions experienced similar rainfall. From AD1600 the pattern reverses and Mapungubwe becomes relatively dry until approximately AD1700 when the two regions experience similar rainfall again. Thereafter Mapungubwe is generally drier than Pafuri. If this were driven by the E/W position of the TTT then the differential should correlate with the Agulhas Current SST, and this is shown to be the case (r = -0.362, p<0.001, n = 197) (Fig 6).

If the position of the TTT system over southern Africa is determined by SST in the Agulhas Current, then the transition from wet conditions during the Medieval Warm Period to dry conditions during the Little Ice Age in Pafuri is unlikely to be the result of this mechanism. Wet conditions are associated with the TTT system in its westward modal position [18] and further westward displacement of the TTT system would lead to reduced rainfall. It is therefore not tenable that the high rainfall during the Medieval Warm Period was driven by TTT modality. Woodborne et al. [2] suggested that this regime shift might be driven by the N/S positions of the subtropical westerlies and the ITCZ. We suggest a northward displacement of the ITCZ at AD 1600–1650. This is consistent with the observation of Cook et al. [36], who note that southward displacements of the ITCZ are associated with wetter conditions in the summer rainfall region of southern Africa. It is also consistent with other palaeodata that suggests that the westerlies expanded northward during the Little Ice Age [37, 38].

### Composite rainfall proxy record

**Fig 7:**
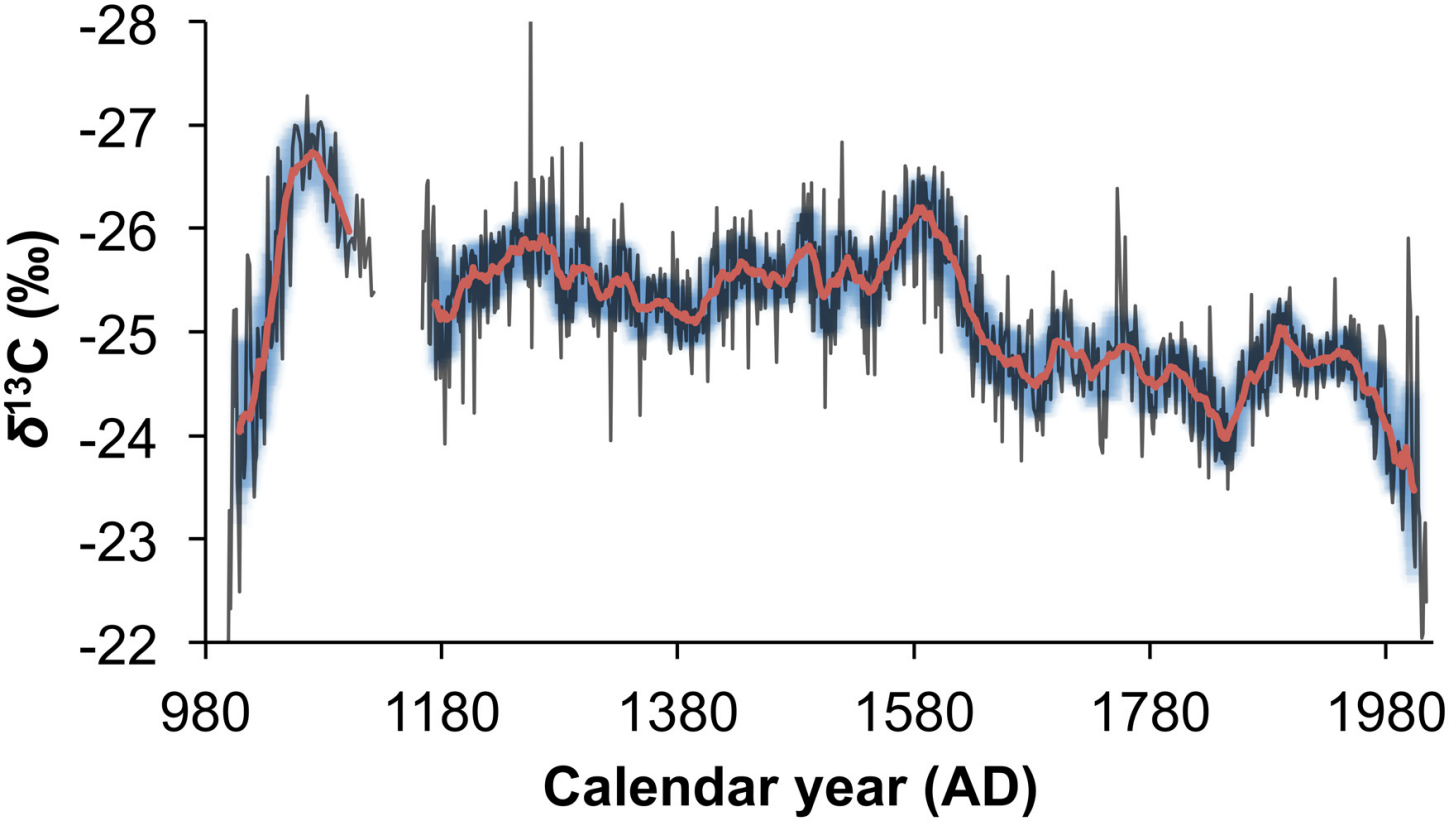
A regional 1000-year rainfall proxy record for the summer rainfall region of South Africa. The isotope proxy record from 4 baobabs in the Pafuri area are combined with that from 5 baobabs from the Mapungubwe area to provide a regional rainfall proxy record for the last 1000 years (black). The 21-year biweight mean (red) and biweight mean variance (blue shading) provide the climate trajectory of the dataset.

The records from Mapungubwe and Pafuri can be combined to provide a single record with higher reproducibility. The combined record and the 21-year biweight mean are presented in Fig 7. The Mapungubwe, revised Pafuri and combined data can be accessed at https://www.ncdc.noaa.gov/paleo/study/19320.

## Conclusions

The age model that was generated for the Mapungubwe baobabs demonstrates that the trees do not necessarily form annual rings, and the age model used to generate the Pafuri record was amended to produce a revised record. The *δ*^13^C record derived from the Mapungubwe area is significantly correlated with the revised Pafuri record. Both proxies correlate with local precipitation and demonstrate a regional shift from wet conditions during the Medieval Warm Period transitioning to dry conditions during the Little Ice Age. A non-stationary residual between the Pafuri and Mapungubwe records is significantly correlated with SST in the Agulhas Current region indicating that localized variation in rainfall is linked to E/W displacement of the temperate tropical trough rainfall system. By combining the two records we enhance the sampling depth, providing a more comprehensive 1000-year rainfall proxy record for the northern summer rainfall region in southern Africa.
